# The Compression Optimality of Asymmetric Numeral Systems

**DOI:** 10.3390/e25040672

**Published:** 2023-04-17

**Authors:** Josef Pieprzyk, Jarek Duda, Marcin Pawłowski, Seyit Camtepe, Arash Mahboubi, Paweł Morawiecki

**Affiliations:** 1 Institute of Computer Science, Polish Academy of Sciences, 01-248 Warsaw, Poland; 2 Data61, CSIRO, Sydney, NSW 2122, Australia; 3 Institute of Computer Science and Computer Mathematics, Jagiellonian University, 30-348 Cracow, Poland; 4 School of Computing and Mathematics, Charles Sturt University, Port Macquarie, NSW 2444, Australia

**Keywords:** entropy coding, source coding, lossless compression, ANS

## Abstract

Source coding has a rich and long history. However, a recent explosion of multimedia Internet applications (such as teleconferencing and video streaming, for instance) renews interest in fast compression that also squeezes out as much redundancy as possible. In 2009 Jarek Duda invented his asymmetric numeral system (ANS). Apart from having a beautiful mathematical structure, it is very efficient and offers compression with a very low coding redundancy. ANS works well for any symbol source statistics, and it has become a preferred compression algorithm in the IT industry. However, designing an ANS instance requires a random selection of its symbol spread function. Consequently, each ANS instance offers compression with a slightly different compression ratio. The paper investigates the compression optimality of ANS. It shows that ANS is optimal for any symbol sources whose probability distribution is described by natural powers of 1/2. We use Markov chains to calculate ANS state probabilities. This allows us to precisely determine the ANS compression rate. We present two algorithms for finding ANS instances with a high compression ratio. The first explores state probability approximations in order to choose ANS instances with better compression ratios. The second algorithm is a probabilistic one. It finds ANS instances whose compression ratios can be made as close to the best ratio as required. This is done at the expense of the number θ of internal random “coin” tosses. The algorithm complexity is $O(\theta L^{3})$, where *L* is the number of ANS states. The complexity can be reduced to $O(\theta L\log_{2}L)$ if we use a fast matrix inversion. If the algorithm is implemented on a quantum computer, its complexity becomes $O(\theta {\left(\log_{2}L\right)}^{3})$.

## 1. Introduction

The increasing popularity of working from home has dramatically intensified Internet traffic. To cope with heavy communication traffic, there are essentially two options. The first option involves an upgrade of the Internet network. This is, however, very expensive and not always available. The second option is much cheaper and employs compression of transmitted symbols. It also makes sense as typical multimedia communication is highly redundant. Source coding has a long history, and it can be traced back to Shannon [1] and Huffman [2]. The well-known Huffman code is the first compression algorithm that works very well for symbol sources, whose statistics follow the natural powers of 1/2. Unfortunately, Internet traffic sources almost never have such simple symbol statistics.

Compression algorithms can be divided into two broad categories: lossy and lossless. Lossy compression does not allow users to recover original data but is widely used for multimedia data such as voice, music, video and picture, where a loss of a few least significant bits does not matter. Ahmed et al. [3] have introduced a lossy compression that applies discrete cosine transform (DCT). The algorithm is now used to compress images (JPEG [4] and HEIF formats), video (MPEG and AVC formats) and music (such as MP3 and MP4 [5,6]). In contrast to lossy compression, a lossless one guarantees the recovery of original data at the receiver side. The lossless compression family includes Huffman code [2] and its variants (see [7] for example), arithmetic coding (AC) [8,9,10], Lempel–Ziv compression and its variants [11,12,13], prediction by partial matching (PPM) [14], run-length encoding (RLE) [15] and, last but not least, asymmetric numeral systems (ANS) [16]. Recent developments in quantum technology bring us closer to the construction of real-life quantum computers [17]. A natural question is whether or not compression of quantum data is feasible [18,19]. The question has been answered in the affirmative in the work [20], which shows how to compress an ensemble of qubits into exponentially fewer qubits. An implementation of quantum compression on IBM quantum computers is reported in the work [21].

ANS introduced by Duda in [16] offers a very versatile compression tool. It allows the compression of symbols that occur with an arbitrary probability distribution (statistics). ANS is also very fast in both hardware and software. Currently, ANS is the preferred compression algorithm in the IT industry. It has been adopted by Facebook, Apple, Google, Dropbox, Microsoft, and Pixar, to name a few main IT companies (for details see https://en.wikipedia.org/wiki/Asymmetric_numeral_systems, accessed on 13 April 2023). ANS can be seen as a finite state machine (FSM) that starts from an initial state, absorbs symbols from an input source one by one and squeezes out binary encodings. The heart of an ANS algorithm is its *symbol spread function* (or simply *symbol spread* for short) that assigns FSM states to symbols. The assignment is completely arbitrary as long as each symbol *s* is assigned a number $L_{s}$ of states such that $p_{s}\approx L_{s}/L$, where $p_{s}$ is probability of the symbol *s* and $L=2^{R}$ is the total number of states (*R* is a parameter that can be chosen to obtain an acceptable approximation of $p_{s}$ by $L_{s}/L$).

Consequently, there are two closely related problems while designing ANS. The first, called *quantisation*, requires from the designer to approximate a symbol probability distribution $P=\{p_{s}|s\in S\}$ by its approximation $Q=\{q_{s}=L_{s}/L|s\in S\}$, where S is the set of all symbols (also called an alphabet). It is expected that ANS implementation for Q achieves a compression ratio that is as close as possible to the symbol source entropy. The second problem is the selection of a symbol spread for fixed ANS parameters. It turns out that some symbol spreads are better than others. Again, an obvious goal is to choose them in such a way that the average encoding length is as small as possible and close to the symbol source entropy.

Motivation. Designers of ANS have to strike a balance between efficiency and compression quality. The first choice that has to be made is how closely symbol probabilities $p_{s}$ need to be approximated by $\frac{L_{s}}{L}$. Clearly, the bigger the number of states ($L=2^{R}$), the better approximation and better compression. Unfortunately, a large *L* slows down compression. It turns out that the selection of a symbol spread has an impact on the quality of compression. For some applications, this impact cannot be ignored. This is true when ANS is applied to build a pseudorandom bit generator. In this case, the required property of ANS is minimal coding redundancy. Despite the growing popularity of ANS, there is no proof of its optimality for arbitrary symbol statistics. This work does not aim to prove optimality, but rather, it aims to develop tools that allow us to compare the compression quality of different ANS instances. It means that we are able to adaptively modify ANS in such a way that every modification provides a compression ratio gain (coding redundancy reduction). The following issues are the main drivers behind this work:

- Investigating symbol quantisation and its impact on compression quality.
- Understanding the impact of a chosen symbol spread on the ANS compression ratio.
- Designing an algorithm that allows us to build ANS instances that maximise compression ratio or equivalently minimises coding redundancy.

Contributions. They are as follows:

- The application of Markov chains to calculate ANS compression ratios. Note that Markov chains have been used in ANS analysis previously. The work [22] applies them for analysis of ANS-based encryption while the paper [23] uses Markov chains to prove the asymptotic optimality of ANS.
- Designing “good” ANS instances whose state probabilities follow the approximation $\log_{2}e/x$, where *x* is an ANS state.
- A randomised algorithm that permits the building of ANS, whose compression ratio is close or alternatively equal to the best possible. The algorithm uses a pseudorandom number generator (PRNG) as a random “coin”.
- An improvement of the Duda–Niemiec ANS cryptosystem that selects at random ANS instances with best compression ratios.

The rest of the work is organised as follows. Section 2 puts the work in the context of the known source coding algorithms. Section 3 describes the ANS compression and its algorithms. Section 4 studies the optimality of ANS. Section 5 shows how Markov chains can be used to calculate ANS state equilibrium probabilities and, consequently, average lengths of ANS encodings. Section 6 presents an algorithm that produces ANS instances whose state probabilities follow the approximation $\log_{2}e/x$. Section 7 describes an algorithm that permits obtaining the best (or close to it) compression ratio. Section 8 suggests an alternative to the Duda–Niemiec ANS encryption. The alternative encryption called cryptographic ANS allows us to design an ANS secret instance whose compression ratio is close to the best one. Section 9 presents the results of our experiments and finally, Section 10 concludes our work.

## 2. Arithmetic Coding versus Asymmetric Numeral Systems

Two variants of ANS (tabled tANS and range rANS) are often used as a direct replacement not only for Huffman coding to improve compression ratio but also for arithmetic coding (AC [8,9,10]) to improve speed. In contrast to Huffman coding (which can handle whole bits only), AC and ANS can process fractions of bits. This requires an additional buffer to store bit fractions and accumulate them until getting a whole bit, which is next released into the output bitstream. AC applies a buffer that stores a range represented by two numbers whose length depends on the probability of encoded symbols. On the other hand, ANS maintains a buffer with a single natural number, whose length reflects both the probability of the encoded symbol and the previous contents of the buffer. In other words, compared to AC, ANS needs a single number only to store the current state.

For both AC and ANS, there are two possible implementation options. The first applies basic arithmetic operations (addition, multiplication and division) performed on large states very close to Shannon entropy. This option has a lower memory cost and is convenient for vectorisation in CPU/GPU architectures. More importantly, it allows for the modification of symbol probabilities on the fly. This option is used in both AC and rANS. The second option translates compression into FSM. It avoids arithmetic operations (especially multiplication) and is recommended for fixed symbol probability distributions. Importantly, it allows for joint compression and encryption. This option is used by quasi-AC, M coder, and tANS.

### 2.1. Arithmetics: AC versus rANS

A classical AC for binary alphabet (bitwise) uses one multiplication per symbol and two for a large alphabet. In contrast, rANS uses one multiplication per symbol no matter how big the alphabet is. This is true for the rANS decoder, as its encoder requires integer division, which is notoriously expensive on most CPU architectures. However, in many applications, decoding speed is more important than encoding speed. One of the fundamental differences between AC and ANS is encoding and decoding orders. For AC, the orders are the same, while for ANS, decoding is executed in reverse. This means that AC is advantageous for some applications, such as streaming. On the other hand, the ANS reverse decoding order enables methods such as the alias method (https://fgiesen.wordpress.com/2014/02/18/rans-with-static-probability-distributions, accessed on 13 April 2023) and the BB-ANS method of Townsend et al. [24], which are not possible with AC. rANS uses a state determined by a single natural number that makes low-level optimisation and vectorisation possible. In contrast, AC has to keep two numbers that define a state and a current range.

In practice, rANS is used for relatively large alphabets ranging from $2^{8}$ to $2^{16}$ symbols, whose probability distribution needs a regular update. For static probability distribution, rANS applies alphabets with 256 symbols. It also copes very well with much larger alphabets with, say, $2^{25}$ symbols [25]. An obvious advantage of a large alphabet used in rANS is a reduction of the required number of steps. Consider two alphabets: one with two and the other with 256 symbols. The number of steps for the larger alphabet can then be reduced by the factor of 8, and there is no need for binary conversion of states. A drawback of rANS with large alphabets is the growing size of tables and an increase in the number of variables. A typical compromise to cope with the dilemma is to deploy byte-oriented arithmetics.

In contrast, AC is usually applied for binary alphabets [26,27]. As we have already pointed out, this creates a significant computational overhead as symbols of large alphabets need to be represented in binary. Fortunately, AC can also work directly with large alphabets. This is referred to as range coding. Note that the implementation of both AC and rANS requires a similar number of operations (with the exception that rANS needs a single multiplication per symbol while AC needs two). However, the main advantage of rANS over AC is a shorter state. A state for AC is determined by two numbers, while a state for rANS consists of a single integer. Moreover, rANS is easier to parallelise due to the fact that the decoder goes through the exact same states as the encoder (albeit in reverse order) [28]. Consequently, rANS low-level implementations can be easily optimised and vectorised (SIMD, GPU).

### 2.2. FSM Representation: Quasi-AC, M Coders and tANS

It turns out that the most expensive arithmetic operation applied in compression is multiplication. To improve efficiency, the idea is to eliminate multiplication altogether. This can be done for a majority of popular entropy coders with a relatively small number of states. As an entropy coder can be seen as FSM, it is enough to determine its state-transition table. An encoding table accepts a pair (symbol, current state) and outputs (binary encoding, next state). Clearly, there must exist a corresponding decoding table that reverses encoding. Quasi-AC is an example of an FSM representation for AC (https://kuscholarworks.ku.edu/bitstream/handle/1808/7210/HoV93.qtfull.pdf;sequence=1, accessed on 13 April 2023). There is also a very popular 64-state M coder ( https://iphome.hhi.de/marpe/mcoder.htm, accessed on 13 April 2023), which is the core of the Context Adaptive Binary Arithmetic Coder (CABAC). The coder is used in MPEG video compressors. As far as we know, all FSM variants of AC are restricted to binary alphabets. Theoretically, larger alphabets can also be converted into their binary equivalents. However, the number of states would be much larger than for ANS as each AC state consists of a pair of numbers (the range). In contrast, thanks to single-number states, ANS can be easily converted into its FSM equivalent for a large alphabet. Table-based ANS (tANS) usually uses from 4 to 8 times more states than the alphabet size. It means that for an alphabet containing 256 symbols, tANS needs 2048 states. This tANS variant is used in a popular FSE implementation of the Zstandard compressor and it rebuilds encoding/decoding tables for every new symbol frame (30 kB in length).

### 2.3. LIFO Handling and Statistical Modelling

ANS works according to the last-in-first-out (LIFO) principle. This technical difficulty is solved by encoding symbols backwards and storing a final state. Then decoding can proceed forward from the final state. If an initial encoding state is fixed and known to a decoder, then the decoder can verify decompression correctness. This way, we obtain a popular checksum mechanism for free. For simple statistical models (like Markov), we can directly encode backwards and use a context of future-forward decoding. For more sophisticated models (like a popular adaptive update of the probability distribution) we can use an additional buffer to store symbol statistics. An encoder first makes a statistical analysis of incoming symbols in their natural (forward) order. It stores the observed probability distribution of symbols in the buffer. Knowing probability distribution, the encoder can next design an appropriate instance of ANS and share it with the decoder. Finally, the encoder processes symbols backwards. The decoder recovers symbols in their natural order. Note that the decoder needs to know where a binary frame terminates. There are essentially the following two options: in the first, we can fix the length of a binary frame; in the second option, we can use a sentinel value that is chosen from a set of low-probability symbols (that do not occur in the current symbol frame).

### 2.4. Industry Status

For AC, there are implementations that achieve compression speeds from ∼50 (alphabet with two symbols) to ∼150 MB/s/core (alphabet with 256 symbols). On the other hand, consider ANS: its compression speed ranges from ∼500 (for FSE implementation of tANS) to ∼1500 MB/s/core (for vectorised SIMD rANS) (see https://github.com/jkbonfield/rans_static, https://sites.google.com/site/powturbo/entropy-coder, accessed on 13 April 2023). For GPU, there are available vectorised rANS implementations (e.g., Facebook or NVidia) reaching speeds of hundreds GB/s (see https://github.com/facebookresearch/dietgpu, https://developer.nvidia.com/blog/latest-releases-and-resources-feb-3-10/#software-releases, accessed on 13 April 2023). To our best knowledge, we are not aware of such fast implementations for AC. There are also available FPGA implementations processing one symbol per cycle [29]. As a consequence, in recent years, ANS has started to dominate the compression market for various applications. Notable examples include compression for:

- General purpose—Facebook Zstandard (tANS) [30] has become probably the most popular replacement of the gzip algorithm (built-in Linux kernel),
- Genetic data—almost the default is CRAM (rANS) citecram, which is a part of the SAMtools library,
- 3D data (meshes, point clouds)—the Google Draco 3D compressor (rANS) used by Pixar,
- Images—JPEG XL (rANS) [31] is recommended by Adobe and often provides the best benchmark performance (https://cloudinary.com/blog/contemplating-codec-comparisons, accessed on 13 April 2023).

The exception is video compression, which is still dominated by AC. This is due to the following two factors: the first one concerns intellectual property and patent issues in relation to the legacy compression algorithms (https://en.wikipedia.org/wiki/Asymmetric_numeral_systems, accessed on 13 April 2023); the second factor is backward encoding required by ANS, which complicates the implementation of compression.

## 3. Asymmetric Numeral Systems

Here we do not describe the ideas behind ANS design. Instead, we refer the reader to original papers by Duda [16,32] and the ANS Wikipedia page, whose URL address is given in the previous section. A friendly introduction to ANS can be found in the work [22]. The ANS algorithm suite includes initialisation, compression (encoding), and decompression (decoding). Algorithm 1 shows initialisation— it also serves as a reference for basic ANS notation.
**Algorithm 1: **ANS Initialisation**Input**: a symbols source S, its symbol probability distribution p:S→[0,1] and a parameter $R\in N^{+}$.**Output**: instantiation of encoding and decoding functions:C(s,x) and $k_{s}\left(x\right)$;D(x) and k(x).**Steps**: proceed as follows:Calculate the number of states $L=2^{R}$;Determine the set of states I={L,…,2L−1};Compute integer $L_{s}\approx Lp_{s}$, where $p_{s}$ is probability of *s*; s∈S;Choose symbol spread function $\bar{s}:I\to S$, where $L_{s}=\left\{x:\bar{s}\left(x\right)=s\right\}$ denotesa set of states assigned to the symbol *s* and $L_{s}=\left|L_{s}\right|$;Establish coding function C(s,y)=x for $y\in \{L_{s},\ldots ,2L_{s}-1\}$,which assigns states $x\in L_{s}$ according to symbol spread function;Compute $k_{s}\left(x\right)=\left\lfloor \log_{2}\left(x/L_{s}\right)\right\rfloor$ for x∈I and s∈S. It givesthe number of output bits per symbol;Construct decoding function D(x)=(s,y), which for x∈I, assignsits unique symbol (given by the symbol spread function) and theinteger *y*, where $L_{s}\leq y\leq 2L_{s}-1$. Note that $D\left(x\right)=C^{-1}\left(x\right)$;Calculate $k\left(x\right)=R-\lfloor \log_{2}\left(y\right)\rfloor$, which determines the number of bitsthat need to be read out from the bitstream;

A compression algorithm accepts a symbol sequence s and outputs a bitstream b. In most cases, the probability distribution of symbols is unknown so it has to be calculated. This is done by pushing symbols one by one to a stack and counting their occurrences. When the last symbol $s_{\ell }$ is processed, the symbol statistics are known, and compression can start. It means that compression starts from the last symbol $s_{\ell }$. Algorithm 2 describes the ANS compression steps.
**Algorithm 2: **ANS Encoding**Input**: a symbol sequence $s=\left(s_{1},s_{2},\ldots ,s_{\ell }\right)\in S^{*}$ and an initial state $x=x_{\ell }\in I$, where ℓ=|s|.**Output**: a bitstream $b=(b_{1}|b_{2}\left|\ldots \right|b_{\ell }{)\in \left\{0,1\right\}}^{*}$, where $|b_{i}|=k_{s_{i}}\left(x_{i}\right)$ and $x_{i}$ is state in *i*-th step.
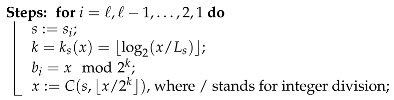
store the final state $x_{0}=x$;

    Note that the bitstream is created by the concatenation of individual encodings. This is denoted by $b=(b_{1}|b_{2}|\ldots |b_{\ell })$, where $b_{i}$ is a binary encoding (sequence of *k* bits); i=1,…,ℓ. Decompression steps are shown in Algorithm 3. Note that $LSB{\left(b\right)}_{k}$ and $MSB{\left(b\right)}_{k}$ stand for the *k* least and most significant bits of b, respectively.
**Algorithm 3: **ANS Decoding**Input**: a bitstream $b\in {\left\{0,1\right\}}^{*}$ and the final state $x=x_{0}\in I$.**Output**: symbol sequence $s\in S^{*}$.
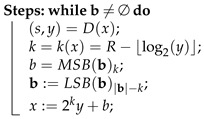


### ANS Example

Given a symbol alphabet S={a,b,c}, where $p_{a}=\frac{3}{16}$, $p_{b}=\frac{5}{16}$, $p_{c}=\frac{8}{16}$ and the parameter R=4. The number of states is $L=2^{R}=16$ and the state set equals to I={16,17,…,31}. A symbol spread $\bar{s}:I\to S$ can be chosen at random as long as (1) the number of elements in $L_{i}$ equals to $L_{i}$ and (2) the chosen states in $L_{i}$ are in the increasing order. So we assume that

(1) $$\begin{array}{ccc} & \bar{s}\left(x\right)=\left\{\begin{array}{cc} a & \mathrm{if}\;x\in \left\{18,22,25\right\}=L_{a}\\ b & \mathrm{if}\;x\in \left\{19,20,23,26,28\right\}=L_{b}\\ c & \mathrm{if}\;x\in \left\{16,17,21,24,27,29,30,31\right\}=L_{c} \end{array}\right. & \\ & ⇕ & \\ & \left.\begin{array}{cccccccccccccccc} 16 & 17 & 18 & 19 & 20 & 21 & 22 & 23 & 24 & 25 & 26 & 27 & 28 & 29 & 30 & 31\\ c & c & a & b & b & c & a & b & c & a & b & c & b & c & c & c \end{array}\right. \end{array}$$

where $L_{a}=3$, $L_{b}=5$ and $L_{c}=8$. The encoding table C(xi,si)=(xi+1,bi)≡def(xi+1bi) is shown in Table 1.

This example is used throughout the work to illustrate our considerations.

## 4. Optimality of ANS—Bounds and Limits

There are two main factors that determine how well ANS compresses symbol sequences: (1) compression quality of the underlying ANS encoder and (2) the length of auxiliary data needed by a decoder to recover symbols from bitstream. Auxiliary data include symbol statistics, the ANS spread function, a chosen sentinel value (or the length of bitstream), and the final ANS state. Note that auxiliary data introduces a loss of compression quality. The loss is significant for relatively short symbol sequences, but it becomes negligible for very long files. In this section, our considerations are focused on ANS compression quality only. We ignore auxiliary data.

Given an ANS instance designed for a memoryless symbol alphabet with its probability distribution $p=\{p_{s};s\in S\}$ and the parameter *R*. The set of all ANS states is $I=\{2^{R},\ldots ,2^{R+1}-1\}$. ANS is optimal if the average length of the binary encoding is equal to the alphabet entropy or

(2) $$\frac{1}{\ell }\sum_{i=1}^{\ell }\left|b_{i}\right|=H\left(S\right)=\sum_{s\in S}p_{s}\log_{2}p_{s}^{-1},$$

where a sequence $s=(s_{1},s_{2},\ldots ,s_{\ell })$ consists of *ℓ* symbols. The sequence s is also called a symbol frame. The same symbols of the frame can be grouped so we get

(3) $$\frac{1}{\ell }\sum_{i=1}^{\ell }|b_{i}|=\frac{1}{\ell }\sum_{s\in S}\sum_{s\in s}\left|b_{s}\right|=\sum_{s\in S}\frac{\sum_{s\in s}\left|b_{s}\right|}{\ell }.$$

Note that $\frac{1}{\ell }\sum_{s\in s}\left|b_{s}\right|=p_{s}\log_{2}p_{s}^{-1}$. So we have proven the following lemma.

**Lemma 1.**  *Given an alphabet S whose probability distribution is $p=\{p_{s};s\in S\}$ and ANS for S. Then ANS is optimal if and only if*

(4) $$\frac{1}{\ell }\sum_{s\in s}\left|b_{s}\right|=p_{s}\log_{2}p_{s}^{-1}for\;all\;s\in S,$$

*where s is a symbol sequence, which repeats every symbol $\ell \cdot p_{s}$ times.*

A caveat—strictly speaking Equation (3) holds when probabilities $p_{s}$ are natural powers of 1/2. In other cases, the left-hand side is a rational number while the right-hand side is an irrational one. Consequently, Equation (3) is an approximation determined by the applied calculation precision.

Let us have a closer look at how ANS encodes symbols. According to Algorithm 2, a symbol *s* is encoded into $b_{i}=x\;\left(\mathrm{mod}\;2^{k}\right)$, where $k_{s}=\left\lfloor \log_{2}\left(x/L_{s}\right)\right\rfloor$ and $x\in \{2^{R},\ldots ,2^{R+1}-1\}$. The length $k_{s}$ of encoding depends on the state *x*. The shortest encoding is when $x=2^{R}$ and the longest when $x=2^{R+1}-1$. It means that

$$\left\lfloor \log_{2}\left(2^{R}/L_{s}\right)\right\rfloor \leq k_{s}\leq \left\lfloor \log_{2}\left(\left(2^{R+1}-1\right)/L_{s}\right)\right\rfloor .$$

As $L_{s}=2^{R}p_{s}$, we get $\left\lfloor \log_{2}p_{s}^{-1}\right\rfloor \leq k_{s}\leq \left\lfloor \log_{2}p_{s}^{-1}\left(2-\frac{1}{2^{R}}\right)\right\rfloor$. Consider a general case when $2^{-(i+1)}<p_{s}<2^{-i}$. Then $k_{s}\in \left\{i,i+1\right\}$. If $p_{s}=2^{-i}$, then the inequality

$$\left\lfloor \log_{2}2^{i}\right\rfloor \leq k_{s}\leq \left\lfloor \log_{2}2^{i}\left(2-\frac{1}{2^{R}}\right)\right\rfloor$$

points to a single encoding length $k_{s}=i$. The above leads us to the following conclusion.

**Lemma 2.**  *Given ANS described by Algorithm 2. Then a symbol s is encoded into a binary string of the length $k_{s}$, where*

- *$k_{s}=i$ if $p_{s}=2^{-i}$,*
- *$k_{s}\in \left\{i,i+1\right\}$ if $2^{-(i+1)}<p_{s}<2^{-i}$. This includes an interesting case when $2^{-1}<p_{s}<2^{0}$ with $k_{s}\in \left\{0,1\right\}$—some symbols are encoded into void bits* ⌀.

Consider a general case of a symbol alphabet S with an arbitrary probability distribution $p=\{p_{s};s\in S\}$ (not necessarily the natural powers of 1/2). Given an ANS instance designed for the distribution *p* and the set of states $I=\{2^{R},\ldots ,2^{R+1}-1\}$. We start from an observation that the average length (the expectation) of binary encoding of a single symbol s∈S into a binary encoding $b_{s}$ is equal to

(5) $$E\left(b_{s}\right)=\frac{1}{R}\sum_{x\in I}p\left(x\right)k_{s}\left(x\right)=\frac{1}{R}\sum_{x\in I}p\left(x\right)\left\lfloor \log_{2}\left(x/L_{s}\right)\right\rfloor ,$$

where a state probability distribution is P={p(x);x∈I}. Note that optimal encoding of s∈S occurs when $|b_{s}|=\log_{2}p_{s}^{-1}$ and does not (directly) depend on the encoding function *C*. It, however, has a direct impact on state probabilities. Assume that we have found an ANS instance which is optimal. This means that each symbol is compressed into its binary encodings whose expected length equal to the symbol entropy. This implies that the following system of equations must hold:

(6) $$\frac{1}{R}\sum_{x\in I}p\left(x\right)k_{s}\left(x\right)=\log_{2}p_{s}^{-1}\mathrm{for}\;s\in S.$$

Consequently, we have proven the lemma presented below.

**Lemma 3.**  *Given an ANS instance designed for the symbol probability distribution $\left\{p_{s};s\in S\right\}$ and the set of states $I=\{2^{R},\ldots ,2^{R+1}-1\}$. Then ANS is optimal if its state probabilities satisfy relations given by Equation (6).*

Let us discuss briefly the consequences of Lemma 3.

- In general, ANS allows us to attain close to optimal compression. However, finding an appropriate coding function *C* seems to be a challenge, especially for a large enough *R*, where enumeration of all possible *C* is not possible. There is also a possibility that there is no such *C*.
- The system given by Equation (6) is underdetermined so its solutions create a linear subspace. To see that this is true, it is enough to observe that for a given symbol, the symbol spread assigns more than one ANS state. Consequently, we have |S| equations in $2^{R}$ variables.
- The work [33] shows how to change the state probabilities so they follow a uniform distribution using a pseudorandom bit generator (PRBG). This interference has been necessary to provide confidentiality and integrity of a compressed stream. Unfortunately, the price to pay is a loss of compression ratio. This approach can be applied here. It would involve a design of PRBG with fine control of probability distribution, which could be a very difficult task. Additionally, running PRBG would be much more expensive than a single call to *C*.
- An optimistic conclusion is that ANS has no structural weaknesses. Whether or not it attains close to optimal compression ratio depends on the coding function *C* or alternatively on the symbol spread.

It is important to make clear the difference between the optimal and the best compression ratios. Given an instance of ANS. We can exhaustively run through all possible symbol spreads and identify the one whose compression ratio is the best. This obviously does not guarantee that such ANS is optimal.

## 5. State Probabilities and Markov Chains

ANS can be looked at as FSM, whose internal state x∈I changes during compression. The behaviour of ANS states is probabilistic and can be characterised by state probability distribution {p(x);x∈I}. For a given symbol s∈S, the average encoding length of *s* is $\kappa \left(s\right)=\sum_{x\in I}k_{s}\left(x\right)p\left(x\right)$, where $k_{s}\left(x\right)$ is the length of an encoding assigned to *s* when ANS is in the state *x* (see Algorithm 2). The average length of ANS encodings is $\kappa =\sum_{s\in S}p_{s}\kappa \left(s\right)$. When we deal with an optimal ANS, then κ=H(S), which also means that $\kappa \left(s\right)=H\left(s\right)=\log_{2}p_{s}^{-1}$ for all s∈S. Typically, compression quality is characterised by a ratio between the length of a symbol sequence and the length of the corresponding bitstream. A better measure from our point of view is coding redundancy, which is defined as ΔH=κ−H(S). The measure has the following advantages:

- Easy identification of an optimal ANS instance when ΔH=0;
- Quick comparison of two ANS instances—a better ANS has a smaller ΔH;
- Fast calculation of the length of a redundant part of bitstream, which is ℓ·ΔH bits, where *ℓ* is the number of symbols in the input sequence.

To determine ΔH for an ANS instance, it is necessary to calculate probability distribution {p(x);x∈I}. It depends on a (random) selection of symbol spread. Note that ANS state transitions are probabilistic and can be described by a matrix. If one of the matrix eigenvalues is equal to λ=1, then the corresponding eigenvector points to an equilibrium probability of the related Markov chain  [23,34,35,36]. Note that the ANS Markov chain is in equilibrium when state probabilities do not change after processing single symbols.

Algorithm 4 shows steps for the construction of a system of linear equations, whose solution gives an equilibrium probability distribution $P_{eq}=\left\{p\left(x\right);x\in I\right\}$. The encoding table $C\left(s,x\right)=x^{\prime }$ shows transition of a state *x* to the next state $x^{\prime }$ while encoding/compressing a symbol *s*.
**Algorithm 4: **Equilibrium of ANS States**Input**: ANS encoding table E(s,x) and symbol probability distribution $\left\{p_{s};s\in S\right\}$.**Output**: probability distribution $P_{eq}=\left\{p\left(x\right);x\in I\right\}$ of ANS Markov chain equilibrium.**Steps**:• initialise $2^{R}\times 2^{R}$ matrix *M* to all zeros except the main diagonal M[i,i]=−1 for $i=0,\ldots ,2^{R}-1$ and the last row, where $M[2^{R}-1,j]=1$ for $j=1,\ldots ,2^{R}$;• create a vector *B* with $2^{R}$ entries with all zero entries except the last entry equal to 1;
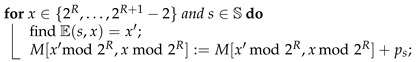
• solve M·X=B using Gaussian elimination;• return *X*→$P_{eq}=\left\{p\left(x\right);x\in I\right\}$;

**Example 1.**  *Consider the ANS instance from Table 1. According to the above algorithm, we obtain a matrix M as follows:*

$$M=\left[\begin{array}{cccccccccccccccc} -\frac{1}{2} & \frac{1}{2} & 0 & 0 & 0 & 0 & 0 & 0 & 0 & 0 & 0 & 0 & 0 & 0 & 0 & 0\\ 0 & -1 & \frac{1}{2} & \frac{1}{2} & 0 & 0 & 0 & 0 & 0 & 0 & 0 & 0 & 0 & 0 & 0 & 0\\ 0 & 0 & -1 & 0 & 0 & 0 & 0 & 0 & \frac{3}{16} & \frac{3}{16} & \frac{3}{16} & \frac{3}{16} & \frac{3}{16} & \frac{3}{16} & \frac{3}{16} & \frac{3}{16}\\ 0 & 0 & 0 & -1 & \frac{5}{16} & \frac{5}{16} & \frac{5}{16} & \frac{5}{16} & 0 & 0 & 0 & 0 & 0 & 0 & 0 & 0\\ 0 & 0 & 0 & 0 & -1 & 0 & 0 & 0 & \frac{5}{16} & \frac{5}{16} & \frac{5}{16} & \frac{5}{16} & 0 & 0 & 0 & 0\\ 0 & 0 & 0 & 0 & \frac{1}{2} & -\frac{1}{2} & 0 & 0 & 0 & 0 & 0 & 0 & 0 & 0 & 0 & 0\\ \frac{3}{16} & \frac{3}{16} & \frac{3}{16} & \frac{3}{16} & 0 & 0 & -1 & 0 & 0 & 0 & 0 & 0 & 0 & 0 & 0 & 0\\ 0 & 0 & 0 & 0 & 0 & 0 & 0 & -1 & 0 & 0 & 0 & 0 & \frac{5}{16} & \frac{5}{16} & \frac{5}{16} & \frac{5}{16}\\ 0 & 0 & 0 & 0 & 0 & 0 & \frac{1}{2} & \frac{1}{2} & -1 & 0 & 0 & 0 & 0 & 0 & 0 & 0\\ 0 & 0 & 0 & 0 & \frac{3}{16} & \frac{3}{16} & \frac{3}{16} & \frac{3}{16} & 0 & -1 & 0 & 0 & 0 & 0 & 0 & 0\\ \frac{5}{16} & \frac{5}{16} & 0 & 0 & 0 & 0 & 0 & 0 & 0 & 0 & -1 & 0 & 0 & 0 & 0 & 0\\ 0 & 0 & 0 & 0 & 0 & 0 & 0 & 0 & \frac{1}{2} & \frac{1}{2} & 0 & -1 & 0 & 0 & 0 & 0\\ 0 & 0 & \frac{5}{16} & \frac{5}{16} & 0 & 0 & 0 & 0 & 0 & 0 & 0 & 0 & -1 & 0 & 0 & 0\\ 0 & 0 & 0 & 0 & 0 & 0 & 0 & 0 & 0 & 0 & \frac{1}{2} & \frac{1}{2} & 0 & -1 & 0 & 0\\ 0 & 0 & 0 & 0 & 0 & 0 & 0 & 0 & 0 & 0 & 0 & 0 & \frac{1}{2} & \frac{1}{2} & -1 & 0\\ 1 & 1 & 1 & 1 & 1 & 1 & 1 & 1 & 1 & 1 & 1 & 1 & 1 & 1 & 1 & 1 \end{array}\right]$$

*The vector B=[0,0,0,0,0,0,0,0,0,0,0,0,0,0,0,1]. The equilibrium probabilities (p(16),…,p(31)) are*

$$\begin{array}{c} \left(\frac{367}{4590},\frac{367}{4590},\frac{1933}{24480},\frac{1189}{14688},\frac{991}{14688},\frac{991}{14688},\frac{367}{6120},\frac{157}{2448}\right.,\\ \left.\frac{1519}{24480},\frac{1189}{24480},\frac{367}{7344},\frac{677}{12240},\frac{367}{7344},\frac{1933}{36720},\frac{157}{3060},\frac{157}{3060}\right). \end{array}$$

*Now it is easy to calculate both the average encoding length κ=1.4790168845 and alphabet entropy H(S)=1.4772170014. ANS leaves a coding redundancy ΔH=κ−H(S)=0.0017998831 bits per symbol.  *

*Let us change the symbol spread by swapping states 25 with 28 so we have the following symbol spread*

$$\bar{s}\left(x\right)=\left.\begin{array}{cccccccccccccccc} 16 & 17 & 18 & 19 & 20 & 21 & 22 & 23 & 24 & 25 & 26 & 27 & 28 & 29 & 30 & 31\\ c & c & a & b & b & c & a & b & c & b & b & c & a & c & c & c \end{array}\right..$$

*The encoding function C(s,y) is:*

$$\begin{array}{cccccccccccccc} s\backslash y & 3 & 4 & 5 & 6 & 7 & 8 & 9 & 10 & 11 & 12 & 13 & 14 & 15\\ a & 18 & 22 & 28 & - & - & - & - & - & - & - & - & - & -\\ b & - & - & 19 & 20 & 23 & 25 & 26 & - & - & - & - & - & -\\ c & - & - & - & - & - & 16 & 17 & 21 & 24 & 27 & 29 & 30 & 31 \end{array}$$

*The encoding table is given in Table 2.*

*After running Algorithm 4, we obtain equilibrium probabilities $\left(p_{16},\ldots ,p_{31}\right)$ as follows:*

$$\begin{array}{c} \left(\frac{3071}{38400},\frac{3071}{38400},\frac{8077}{102400},\frac{4981}{61440},\frac{4177}{61440},\frac{4177}{61440},\frac{3071}{51200},\frac{65}{1024},\right.\\ \left.\frac{6321}{102400},\frac{3071}{61440},\frac{3071}{61440},\frac{17159}{307200},\frac{4981}{102400},\frac{5419}{102400},\frac{13}{256},\frac{13}{256}\right). \end{array}$$

*The average encoding length κ=1.4789314193. This ANS instance leaves coding redundancy ΔH=κ−H(S)=0.0017144179 bits per symbol. Clearly, it offers better compression than the first variant.*

The following remarks summarise the above discussion:

- The extensive practical experience and asymptotic optimality of ANS (see [23]) justifies the conclusion that ANS offers a very close-to-optimal compression when the parameter *R* is big enough so $L_{s}=2^{R}p_{s}$ for all s∈S. In general, however, for arbitrary symbol statistics, ANS tends to leave coding redundancy ΔH≠0.
- In some applications where ANS compression is being used heavily (for instance, video/teleconference streaming), it makes sense to optimise ANS so ΔH is as small as possible. Note that the smaller ΔH, the more random the bitstreams. This may increase the security level of systems that use compression (for instance, joint compression and encryption).
- It is possible to find the best ANS instance by an exhaustive search through all distinct symbol spreads. For $L=2^{R}$ states, we need to search through $\frac{L!}{\prod_{s\in S}L_{s}!}$ ANS instances. This is doable for a relatively small number of states. For ANS in our example, the search space includes $\frac{16!}{8!\cdot 5!\cdot 3!}=720720$ instances. For a bigger *L* (say, above 50), an exhaustive search is intractable.

## 6. Tuning ANS Symbol Spreads

For the compression algorithms such as Zstandard and LZFSE, a typical symbol alphabet contains n=256 elements. To obtain a meaningful approximation of the alphabet statistics, we need a bigger number *L* of states. A rough evaluation of compression ratio penalty given in [37] tells us that choosing L=2n incurs a entropy loss of ΔH=0.01 bits/symbol; if L=4n, then ΔH=0.003 and if L=8n, then ΔH=0.0006. This confirms an obvious intuition that the bigger number of states *L*, the better approximation and, consequently, compression ratio. However, there is a “sweet spot” for *L*, where its further increase slows compression algorithm but without a noticeable compression ratio gain.

Apart from approximation accuracy of symbol probabilities so $p_{s}\approx L_{s}/L$, the symbol spread $\bar{s}:I\to S$ has an impact on compression ratio. Intuitively, one would expect that symbol spread is chosen uniformly at random from all $\frac{L!}{\prod_{s\in S}L_{s}!}$ possibilities. It is easy to notice that they grow exponentially with *L*. An important observation is that the probability distribution of ANS states during compression is not uniform. In fact, a state x∈I occurs with probability that can be approximated as $p\left(x\right)\approx \log_{2}\left(e\right)/x$. Note that this is beneficial for a compression ratio, as smaller states (with shorter encodings) are preferred over larger ones (with longer encodings). The natural probability bias of states has an impact on the compression ratio, making some symbol spreads better than the others. Let us take a closer look at how to choose symbol spread so it maintains the natural bias.

Recall that for a given symbol s∈S and state x∈I, the encoding algorithm (see Algorithm 2) calculates $k=k_{s}=\left\lfloor \log_{2}\frac{x}{L_{s}}\right\rfloor$, extracts *k* least significant bits of the state as the encoding of *s* and finds the next state $x^{\prime }=C\left(s,\left\lfloor \frac{x}{2^{k}}\right\rfloor \right)$, where C(s,y) is equivalent to a symbol spread $\bar{s}$ and $x^{\prime }\in L_{s}$. Consider properties of coding function C(s,y) that are used in our discussion.

**Fact 1.**  *Given a symbol s and coding function $C(s,\left\lfloor \frac{x}{2^{k}}\right\rfloor )$. Then the collection of states I is divided into $L_{s}$ state intervals $I_{i}$, where*

- *Each interval $I_{i}$ consists of all consecutive states that share the same value $\left\lfloor \frac{x}{2^{k}}\right\rfloor$ for all $x\in I_{i}$,*
- *Coding function assigns the same state $x^{\prime }=C\left(s,\left\lfloor \frac{x}{2^{k}}\right\rfloor \right)$ for $x\in I_{i}$ and $x^{\prime }\in L_{s}$,*
- *The cardinality of $I_{i}$ is $2^{k}$, where $k=k_{s}=\left\lfloor \log_{2}\frac{x}{L_{s}}\right\rfloor$ and $I=\cup_{i=1,\ldots ,L_{s}}I_{i}$.*

**Example 2.**  *Take into account ANS described in Table 1. For the symbol a, the collection of states I={16,…,31} splits into $L_{a}=3$ intervals as follows:*

$I_{1}=\left\{16,17,18,19\right\}\;$



$\;I_{2}=\left\{20,21,22,23\right\}\;$



$\;I_{3}=\left\{24,25,26,27,28,29,30,31\right\}\;$

$C(s,\left\lfloor \frac{x}{4}\right\rfloor )=22$; $x\in I_{1}$$\;C(s,\left\lfloor \frac{x}{4}\right\rfloor )=25$; $x\in I_{2}$$\;C(s,\left\lfloor \frac{x}{8}\right\rfloor )=18$; $x\in I_{3}$

*Note that cardinalities of $I_{i}$ are powers of 2 or $|I_{1}\left|=\right|I_{2}|=4$ and $|I_{3}|=8$. As the encoding function C(s,·) is constant in the interval $I_{i}$, it makes sense to use a shorthand $C(s,I_{i})$ instead of $C(s,\left\lfloor \frac{x}{2^{k}}\right\rfloor )$ for $x\in I_{i}$. *

Assume that we know probabilities p(x) of states of x∈I, then the following conclusion can be derived from Fact 1.

**Corollary 1.**  *Given an ANS instance defined by Algorithms 1–3. Then for each symbol s∈S, state probabilities have to satisfy the following relations:*

(7) $$p_{s}\sum_{x\in I_{i}}p\left(x\right)=p\left(C\left(s,I_{i}\right)\right);\;\;\;i=1,\ldots ,L_{s}.$$

Recall that those state probabilities can be approximated by $p\left(x\right)\approx \log_{2}\left(e\right)/x$. As Equation (7) requires summing up probabilities of consecutive states, we need the following fact.

**Fact 2.**  *Given an initial part of the harmonic series $\left(1+\frac{1}{2}+\ldots +\frac{1}{r}\right)$, then it can be approximated as shown below*

(8) $$\sum_{i=1}^{r}\frac{1}{i}=1+\frac{1}{2}+\ldots +\frac{1}{r}\approx \ln\left(r\right)+\gamma ,$$

*where the constant γ≈0.577. It is easy to obtain an approximation of the series $\left(\frac{1}{r}+\ldots +\frac{1}{r+\alpha }\right)$, which is $\approx \ln\left(r+\alpha \right)-\ln\left(r-1\right)=\ln\left(\frac{r+\alpha }{r-1}\right)$, where $\alpha \in N^{+}$.*

Now, we are ready to find out a preferred state for our symbol spread. Let us take into account Equation (7). Using the above established facts and our assumed approximation $p\left(x\right)\approx \log_{2}\left(e\right)/x$, the left-hand side of the equation becomes

$$p_{s}\sum_{x\in I_{i}}p\left(x\right)=p_{s}\log_{2}\left(e\right)\sum_{x\in I_{i}}\frac{1}{x}=p_{s}\log_{2}\left(e\right)\ln\frac{r+\alpha -1}{r-1},$$

where *r* is the first state in $I_{i}$ and (r+α−1)—the last and $\alpha =2^{k}$. As we have assumed that the state $C\left(s,I_{i}\right)\approx \log_{2}\left(e\right)\frac{1}{y}$, where *y* points the preferred state that needs to be included into $L_{s}$, we obtain

$$p_{s}\log_{2}\left(e\right)\ln\frac{r+\alpha -1}{r-1}=\log_{2}\left(e\right)\frac{1}{y}.$$

This brings us to the following conclusion.

**Corollary 2.**  *Given ANS as defined above, then a preferred state y for $I_{i}$ determined for a symbol s is*

(9) $$y={\left(p_{s}\ln\frac{r+\alpha -1}{r-1}\right)}^{-1},$$

*where $I_{i}=\left[r,\ldots ,r+\alpha -1\right]$ and ⌊y⌉ an integer closest to y and it is added to $L_{s}$.*

Algorithm 5 shows an algorithm for the calculation of symbol spread with preferred states.
**Algorithm 5: **Symbol Spread Tuning**Input**: ANS number of states $L=2^{R}$ and symbol probability distribution $\left\{p_{s};s\in S\right\}$.**Output**: symbol spread $\bar{s}$ determined by $L_{s}$ for s∈S.**Steps**: initialise $L_{s}=⌀$ for s∈S;
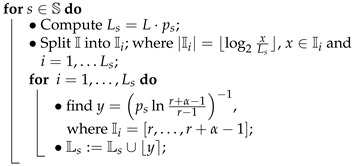
• Remove collisions from $L_{s}$ so $L_{s}\cap L_{s^{\prime }}=⌀$ for $s\neq s^{\prime }$;• Return $\bar{s}$ or equivalently $L_{s}$ for s∈S;

    Let us consider the following example.

**Example 3.**  *Take ANS from Section 3 with L=16 states and three symbols S={a,b,c} that occur with probabilities 3/16, 5/16 and 8/16, respectively. For the symbol a, the set of states I splits into $L_{a}=16p_{a}=3$ subsets: $I_{1}=\left\{16,17,18,19\right\}$, $I_{2}=\left\{20,21,22,23\right\}$ and $I_{3}=\left\{24,\ldots ,31\right\}$. Let us compute the preferred state y for $I_{1}$. It is $y={\left(p_{s}\ln\frac{r+\alpha -1}{r-1}\right)}^{-1}\approx 22.56$, where r=16 and r+α−1=19. For $I_{2}$, we obtain y≈27.91. For $I_{3}$, we obtain y≈17.86. After rounding to the closest integers, $L_{a}=\left\{18,23,28\right\}$. In similar way, we calculate $L_{b}=\left\{17,20,23,26,29\right\}$ and $L_{c}=\left\{16,18,20,22,24,26,28,30\right\}$. Clearly, there are a few collisions, for example, the state 18 belongs to both $L_{a}$ and $L_{c}$. They need to be removed. We accept $L_{c}$. The colliding states in other sets are replaced by their closest neighbours, which are free. This obviously makes sense as neighbour states share similar probabilities. The final symbol spread is as follows*

$$\begin{array}{cccccccccccccccc} 16 & 17 & 18 & 19 & 20 & 21 & 22 & 23 & 24 & 25 & 26 & 27 & 28 & 29 & 30 & 31\\ c & b & c & a & c & b & c & b & c & a & c & b & c & b & c & a \end{array}\;$$

*We can compute the average encoding length by computing equilibrium probabilities, which for the above symbol spread, is $\kappa =\frac{3619}{2448}\approx 1.4783$. It turns out that this is the best compression ratio, as argued in Section 7. In contrast, the average lengths of symbol spreads in Example 1 are 1.4790 and 1.4789.*

Algorithm 5 may produce many symbol spreads with slightly different compression ratios. Preferred positions need to be rounded to the closest integers. Additionally, there may be collisions in sets $L_{s}$ (s∈S) that have to be removed. Intuitively, we are searching for a symbol spread that is the best match for preferred positions. In other words, we need to introduce an appropriate distance to measure the match.

**Definition 1.**  *Given ANS with a symbol spread $L_{s\in S}$ and a collection of preferred positions $L_{s\in S}$ calculated according to Equation (9). Then the distance between them is computed as*

(10) $$d\left(L_{s\in S},L_{s\in S}\right)=\sum_{s\in S}\sum_{x\in L_{s}}\left|x-y\right|,$$

*where y is the preferred position that is taken by x.*

Finding the best match for a calculated $L_{s\in S}$ is equivalent to identification of a symbols spread $L_{s\in S}^{*}$ such that

(11) $$d\left(L_{s\in S}^{*},L_{s\in S}\right)=\min_{L_{s\in S}}d\left(L_{s\in S},L_{s\in S}\right),$$

where the symbol spread $L_{s\in S}$ runs through all possibilities.

Algorithm 6 illustrates a simple and heuristic algorithm for finding $L_{s\in S}^{*}$.
**Algorithm 6: **Finding Symbol Spread $L_{s\in S}^{*}$**Input**: ANS number of states $L=2^{R}$, symbol probability distribution $\left\{p_{s};s\in S\right\}$ and $L_{s\in S}$**Output**: symbol spread $\bar{s}$ determined by $L_{s}^{*}$ for s∈S**Steps**: Create a table with three rows and $L=2^{R}$ columns;Put all consecutive states (i.e., (L,L+1,…,2L−1) inincreasing order in the first row;Insert all numbers from $L_{s\in S}$ in increasing order in the second row together with theirsymbol labels in the third row;Read out all states from the first row that correspond to appropriate symbol labels (in thethird row);Return $\bar{s}$ or equivalently $L_{s}^{*}$ for s∈S;

**Example 4.**  *Consider again the ANS from Section 3 with L=16 states and three symbols S={a,b,c} that occur with probabilities 3/16, 5/16, and 8/16, respectively. We follow Algorithm 6 and obtain the following table:*

$$\begin{array}{ccccccccccccccccc} x & 16 & 17 & 18 & 19 & 20 & 21 & 22 & 23 & 24 & 25 & 26 & 27 & 28 & 29 & 30 & 31\\ y & 16.7 & 16.9 & 17.8 & 17.9 & 19.9 & 19.9 & 21.9 & 22.5 & 23.1 & 23.9 & 25.5 & 25.9 & 27.9 & 27.9 & 28.7 & 29.9\\ s & b & c & a & c & b & c & c & a & b & c & b & c & a & c & b & c \end{array}$$

*After the calculation of equilibrium probabilities, we obtain the average encoding length, which is $\kappa =\frac{230755}{156048}\approx 1.4787$.*

The following observations are relevant:

- The algorithm for tuning symbol spreads is very inexpensive and can be easily applied for ANS with a large number of states (bigger than $2^{10}$). It gives a good compression ratio.
- Equation (9) gives a rational number that needs to be rounded up or down. Additionally, preferred states pointed by it are likely to collide. Algorithm 5 computes a symbol spread, which follows the preferred positions.
- Equation (9) indicates that preferred states are likely to be uniformly distributed over I.
- It seems that finding $L_{s\in S}^{*}$ such that $d(L_{s\in S}^{*},L_{s\in S})$ attains minimum does not guarantee the best compression ratio. However, it results in an ANS instance with a “good” compression ratio. This could be a starting point to continue searching for ANS with a better compression ratio.

## 7. Optimisation of ANS

### 7.1. Case Study

**Figure 1:**
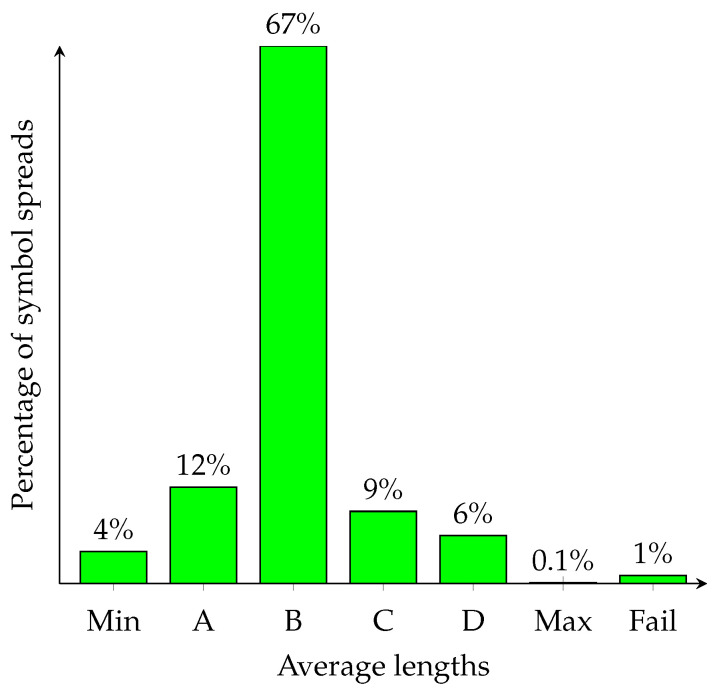
Distribution of average lengths of ANS encodings for the toy ANS. Note that H(S)≈1.477 Legend: A=〈Min,1.48], B=〈1.48,1.49], C=〈1.49,1.5], D=〈1.5,Max〉, $Min=\frac{3619}{2448}\approx 1.478$ and $Max=\frac{97}{64}\approx 1.515$.

Consider a toy instance of ANS with three symbols that occur with probabilities {3/16,5/16,8/16} and 16 states (i.e., R=4). We have implemented software in PARI/GP ( PARI is a free software developed by Henri Cohen. PARI stands for Pascal ARIthmetic and GP—Great Programmable calculator.) that searches through all possible instances of ANS symbol spreads (there are $\frac{16!}{8!\cdot 5!\cdot 3!}=720,720$ such instances). For each spread, we have calculated equilibrium probabilities for the corresponding Markov chain. This allows us to compute the average length of a symbol (in bits/symbol). The results are shown in Figure 1.

Unfortunately, there is a small fraction of ANS instances whose equilibrium probabilities are impossible to establish as the corresponding system is linearly dependent (its rank is 15). An example of symbol spread with the shortest average encoding length is:

$$\bar{s}\left(x\right)=\left\{\begin{array}{cc} a & \mathrm{if}x\in \left\{16,24,25\right\}=L_{a}\\ b & \mathrm{if}x\in \left\{17,20,21,26,27\right\}=L_{b}\\ c & \mathrm{if}x\in \left\{18,19,22,23,28,29,30,31\right\}=L_{c} \end{array}\right.$$

In contrast, the following symbol spread function has the longest average encoding length:

$$\bar{s}\left(x\right)=\left\{\begin{array}{cc} a & \mathrm{if}x\in \left\{24,25,26\right\}=L_{a}\\ b & \mathrm{if}x\in \left\{27,28,29,30,31\right\}=L_{b}\\ c & \mathrm{if}x\in \left\{16,17,18,19,20,21,22,23\right\}=L_{c} \end{array}\right.$$

Clearly, a careful designer of ANS is likely to use a pseudorandom number generator to select symbol spreads. There is better than 1/2 probability that such an instance has the average encoding length somewhere in the interval 〈1.48,1.49]. On the other hand, a careless designer is likely to fall into a trap and choose one of the worst symbol spreads.

### 7.2. Optimal ANS for Fixed ANS Parameters

The idea is to start from a random symbol spread. Then, we continue swapping pairs of ANS states. After each swap, we calculate the coding redundancy of a new ANS instance. If its redundancy is smaller than the old instance redundancy, then we keep the change. Otherwise, we select a new pair of states for the next swap. Details are given in Algorithm 7.
**Algorithm 7: **Search for Optimal ANS**Input**: symbol probability distribution $\left\{p_{s};s\in S\right\}$ and parameter *R* such that $L_{s}=p_{s}2^{R}$ for all *s***Output**: symbol spread $\bar{s}$ or encoding table E(s,x) of ANS with the smallest coding redundancy**Steps**:• Initialise symbol spread $\bar{s}$ using PRBG;• Determine the corresponding E(s,x) and calculate its redundancy ΔH;• Assume a required minimum redundancy threshold *T*;
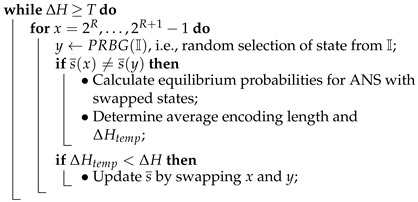
• Return $\bar{s}$ and ΔH;

    We have implemented the algorithm in the PARI/GP environment. Our experiment is executed for the 16-state ANS and three symbols that occur with probabilities {3/16,5/16,8/16}. As the algorithm is probabilistic, its behaviour varies depending on specific coin tosses that determine state swaps. Our starting symbol spread is a spread with the longest average encoding length, i.e., {24,25,26}, {27,28,29,30,31}, {16,17,18,19,20,21,22,23}). This is the worst case.

The algorithm continues to swap state pairs until the average length equals the minimum $\frac{3619}{2448}$ (the best compression ratio). We have run the algorithm $10^{5}$ times. The results are presented in Table 3. The main efficiency measure is the total number of swaps of ANS state pairs that is required in order to achieve the best compression ratio. Note that each state swap forces the algorithm to redesign ANS. As the algorithm uses PRBG, the number of state swaps varies. The algorithm works very well and successfully achieves the best compression ratio every time. In Table 3, we introduce “good swaps”. It means that any good swap produces an ANS instance whose average encoding length gets smaller. This also means that in the worst case, we need only 19 swaps to produce the optimal ANS. Optimality here is understood as the minimum coding redundancy.

The complexity of Algorithm 7 is $O(\theta L^{3})$, where θ is the number of iterations of the main loop of the algorithm. The most expensive part is the Gaussian elimination needed to find equilibrium probabilities. It takes $O(L^{3})$ steps. We assume that we need θ swaps to obtain a required redundancy with high probability. In other words, the algorithm becomes very expensive when the number of states *L* is bigger than $2^{10}$. This is unfortunate, however, the good news is that a system of linear equations defining equilibrium probabilities is sparse. Consider a simple geometric symbol probability distribution {1/2,1/4,…,1/1024,1/1024}. Assume that ANS has $L=2^{10}$ states. A simple calculation indicates that the matrix *M* in the relation M·X=B for the Markov equilibrium has ≈99% zeros. It means that we can speed up the search for the optimal ANS in the following way:

- Use specialised algorithms for Gaussian elimination that targets sparse systems. There are some mathematical packages (such as MatLab, for example) that include such algorithms;
- Apply inversion of sparse matrices (such as the algorithm from [38], whose complexity is $O(L^{2.21})$). First we solve M·X=B by computing $M^{-1}$. This allows us to find *X*. Now we swap two states that belong to different symbols. Now we need to solve $\tilde{M}\cdot \tilde{X}=B$, where $\tilde{M}$ is a system that describes equilibrium after the swap. We build ${\tilde{M}}^{-1}$. Now we translate M·X=B by first I·M·X=B and then $\tilde{M}{\tilde{M}}^{-1}M\cdot X=B$, which produces $\tilde{M}\cdot \tilde{X}=B$, where $\tilde{X}={\tilde{M}}^{-1}M\cdot X$. In other words, the solution $\tilde{X}$ can be obtained from *X* by multiplying it by ${\tilde{M}}^{-1}M$.

Further efficiency improvements can be achieved by taking a closer look at swapping operations. For the sake of argument, consider a swap of two states and their matrices *M* and $\tilde{M}$ that describe their Markov chain probabilities before and after the swap, respectively. There are essentially two distinct cases when the swap is:

- Simple, i.e., only two rows of *M* are affected by the swap. The matrix entries outside the main diagonal are swapped, but entries on the main diagonal need to be handled with care as they do not change if their values are −1. For instance, take ANS from Table 1. Let us swap the states 25 and 26. The rows of *M* before swap are
  $$\begin{array}{ccccccccccccccccc} & 16 & 17 & 18 & 19 & 20 & 21 & 22 & 23 & 24 & 25 & 26 & 27 & 28 & 29 & 30 & 31\\ 25\to & 0 & 0 & 0 & 0 & \frac{3}{16} & \frac{3}{16} & \frac{3}{16} & \frac{3}{16} & 0 & -1 & 0 & 0 & 0 & 0 & 0 & 0\\ 26\to & \frac{5}{16} & \frac{5}{16} & 0 & 0 & 0 & 0 & 0 & 0 & 0 & 0 & -1 & 0 & 0 & 0 & 0 & 0 \end{array}$$
  The rows after the state swap are
  $$\begin{array}{ccccccccccccccccc} & 16 & 17 & 18 & 19 & 20 & 21 & 22 & 23 & 24 & 25 & 26 & 27 & 28 & 29 & 30 & 31\\ 25\to & \frac{5}{16} & \frac{5}{16} & 0 & 0 & 0 & 0 & 0 & 0 & 0 & -1 & 0 & 0 & 0 & 0 & 0 & 0\\ 26\to & 0 & 0 & 0 & 0 & \frac{3}{16} & \frac{3}{16} & \frac{3}{16} & \frac{3}{16} & 0 & 0 & -1 & 0 & 0 & 0 & 0 & 0 \end{array}$$
  The matrix $\tilde{M}$ after swap can be obtained by $\tilde{M}=M\Delta$, where Δ is a sparse matrix that translates *M* into $\tilde{M}$. As argued above, the equilibrium solution is $\tilde{X}=\Delta^{-1}X$, where $\Delta^{-1}={\tilde{M}}^{-1}M$. We still need to calculate ${\tilde{M}}^{-1}$; however, it is possible to recycle a part of the computations performed while computing $M^{-1}$. This is possible as *M* and $\tilde{M}$ share the same entries (except the two rows that correspond to the state swap),  
- Complex, i.e., more than two rows need to be modified. This occurs when a swap causes a cascade of swaps that are needed to restore the increasing order in their respective $L_{s}$. For example, consider ANS from Table 1 and swap the states 22 and 26.
  $$\begin{array}{ccc} & \begin{array}{c} L_{1}=\left\{18,22,25\right\}\\ L_{2}=\left\{19,20,23,26,28\right\} \end{array} & \\ & ↓22↔26 & \\ & \begin{array}{c} \begin{array}{c} \left\{18,26,25\right\}\\ \left\{19,20,23,22,28\right\}\\ \mathrm{invalid}\mathrm{ANS} \end{array} \end{array} & \\ & ↓\begin{array}{c} 25↔26\\ 22↔23 \end{array} & \\ & \begin{array}{c} \left\{18,25,26\right\}\\ \left\{19,20,22,23,28\right\} \end{array} \end{array}$$
  To obtain a valid ANS instance, we need two extra swaps. The calculation of ${\tilde{M}}^{-1}$ can still be supported by the computations for $M^{-1}$ but the matrices *M* and $\tilde{M}$ differ on more than two rows.

### 7.3. Optimalisation with Quantisation

So far we have assumed that $L_{s}=p_{s}\cdot L$; s∈S or at least $L_{s}/L$ is a very close approximation of $p_{s}$. However, this is not always true. In practice, there are two issues that need to be dealt with.

- The first is the fact that $p_{s}\cdot L$ may have two “good” approximations when $\alpha_{s}<p_{s}L<\left(\alpha_{s}+1\right)$, where $\alpha_{s}\in N^{+}$. So, we can choose $L_{s}\in \left\{\alpha_{s},\alpha_{s}+1\right\}$. This occurs more frequently when the number *L* is relatively small.
- The second issue happens when there is a tail of symbols whose probabilities are small enough so $p_{s}\cdot L<1$. It means that symbols have to be assigned to single states $L_{s}=1$. This is equivalent to an artificial increase of symbol probabilities of the tail to 1/L. Note that $\sum_{s\in S}p_{s}=1$, which is equivalent to $\sum_{s\in S}L_{s}=L$. Consequently, the number of states in the symbol spread for other symbols has to be reduced.

The research question in hand (also called quantisation) is defined as follows. Given that a symbol probability distribution $S=\{p_{s}|s\in S\}$, we have to ask: How do we design ANS so its compression is optimal (or close to it), where ANS is built for the symbol probability distribution $Q=\{q_{s}|s\in S\}$, where Q approximates S, where $L_{s}=q_{s}\cdot L\in N^{+}$, $q_{s}\approx p_{s}$ and n=|S|?  

Let us consider the following algorithms.

- Exhaustive search through all possible quantised ANS, where an ANS instance is determined by a selection of $L_{s}\leftarrow \left\{\alpha_{s},\alpha_{s}+1\right\}$, where s∈S. For each selection, we run Algorithm 7. Finally, we choose the ANS instance with the best compression ratio. Unfortunately, the complexity of the algorithm is exponential or more precisely, $O(2^{n}\theta L^{3})$. A possible tail of *t* symbols reduces complexity as all *t* symbols are assigned a single state and the next *t* low probability symbols are assigned $\alpha_{s}$ states. Higher $L_{s}=\alpha_{s}+1$ are ignored in order to compensate states that have been taken by the tail symbols. Note also that some of the selections for $L_{s}$ must be rejected if $\sum_{s\in S}L_{s}\neq L$.
- Best-fit quantised ANS. The idea is to start from symbols from the tail, where $L_{s}$ has to be 1. Symbols with high probabilities are assigned $L_{s}=\alpha_{s}+1$, while symbols with low probabilities $L_{s}=\alpha_{s}$. Symbols with mid-range probabilities are randomly assigned $L_{s}\in \left\{\alpha_{s},\alpha_{s}+1\right\}$. The intuition behind the algorithm is the fact that selection of higher $L_{s}$ reduces the average length of encodings and vice versa. Now we can run Algorithm 7 to find the optimal (or close to it) ANS.

To recap our discussion, we make the following remarks:

- Finding ANS with the optimal compression ratio is of prime concern to anyone who would like to either maximise communication bandwidth or remove as much redundancy as possible from bitstreams. We model the asymptotic behaviour of ANS by Markov chains. The calculated equilibrium probabilities allow us to precisely determine the average length of binary encodings and, consequently, the ANS compression ratio.
- Search for the best ANS can be done in two steps. (1) We run Algorithm either 5 or  6 that tunes symbol spread using an approximation of state probabilities. The algorithm is very efficient, and its complexity is O(L). Unfortunately, it does not guarantee that the calculated ANS instance is optimal/best. (2) Next, we execute Algorithm 7. Its initial symbol spread has been calculated in the previous step. The randomised algorithm usually attains the best (or close to it) ANS in practice (see experiments in Section 9).
- Algorithm 7 can be sped up by using a specialised sparse matrix inversion algorithm together with reusing computations from previous inversions. This allows us to find a close-to best ANS for the number *L* of states in the range $\left[2^{10},2^{12}\right]$. The range is the most used in practice.
- The number of iterations θ contributes to the complexity of Algorithm 7. In general, the bigger θ, the higher the probability that the algorithm produces the best ANS. In fact, θ=αL is enough to obtain ANS with a close to the best compression ratio with high probability, where α is a small integer (say 2 or 3) —see our experiments in Section 9.

## 8. Cryptographic ANS

Duda and Niemiec [39] have proposed a randomised ANS, where its symbols spread is chosen at random. To make it practical, the authors suggest replacing truly random coin tosses with a pseudorandom number generator (PRNG), which is controlled by a relatively short random seed/key. The two communicating parties can agree on a common secret key *K*. Both sender and receiver use it to select their symbol spread using PRNG controlled by *K*. The sender can build an appropriate encoding table, while the receiver—the matching decoding table. Consequently, the parties can use compression as encryption. Although such encryption does not provide a high degree of protection (especially against integrity and statistics attacks—see [22,33]), it could be used effectively in low-security applications. The price to pay is a complete lack of control over the compression ratio of ANS. This weakness can be mitigated by applying our Algorithm 7.

Note that the symbol spread $\left\{L_{s}|p_{s}\in S\right\}$ is public but $\left\{L_{s}^{best}|p_{s}\in S\right\}$ is secret as to reconstruct it, an adversary needs to recover *K* and execute Algorithm 7. The cryptographic ANS is illustrated in Table 4. Unlike the Duda–Niemiec ANS, it achieves a close to the best compression ratio. However, the effective security level (the length of the cryptographic key) is determined by the number of ANS instances produced by Algorithm 7. For instance, the ANS from Figure 1 guarantees 15-bit security (or $\log_{2}30240\approx 15$). For ANS with a large number of states, it is difficult to determine a precise security level. However, this may be acceptable for low-security applications.

## 9. Experiments

Algorithm 7 has been implemented using the Go language and has been executed on a MacBook Pro with an M1 chip. The algorithm has been slightly modified so it finds both the lower and upper bounds for ΔH. The lower bound points to ANS, which is close to the best. In contrast, the upper bound shows ANS, whose coding redundancy ΔH is big (close to the worst case). Note that a random selection of spreads produces ANS instances whose ΔH fall somewhere between the bounds. The following results have been obtained for $10^{5}$ iterations of the FOR loop.
# ANS States1282565121024$\Delta H_{min}$1.5770 $\times 10^{-5}$4.1869 $\times 10^{-6}$1.0470 $\times 10^{-6}$2.7868 $\times 10^{-7}$$\Delta H_{max}$0.03460.02980.03480.0306# Spreads with $\Delta H_{min}$2268138156# Spreads with $\Delta H_{max}$1493577011044Search Time for $\Delta H_{min}$1 m 12 s8 m 30 s1 h 11 m 37 s9 h 2 m 59 sSearch Time for $\Delta H_{max}$1 m 23 s8 m 13 s1 h 9 m 58 s8 h 47 m 5 s

We have increased the number of iterations of the FOR loop to $n^{2}$, where *n* is the number of states for n={512,1024}. The results are presented below.
*n*5121024# Iterations262,1441,048,576# Good Swaps508792$\Delta H_{min}$1.0222607274 $\times 10^{-6}$2.5842 $\times 10^{-7}$$\Delta H_{min}$ after additional rounds1.0222607271 $\times 10^{-6}$2.5842 $\times 10^{-7}$# Additional Rounds$\;10^{6}$$\;10^{5}$Better Spreads Found in Additional Rounds80Execution Time872 m6115 m

We see that due to the probabilistic nature of the Algorithm 7, even after a large number of iterations, there is a non-zero chance of finding a spread with lower ΔH. In practise, there are time restrictions imposed on the time needed for the execution of Algorithm 7. The following results illustrate how much time is needed between two consecutive good swaps that improve ΔH. The number of iterations of the FOR loop is $10^{5}$.
# ANS States1282565121024Tunning Time54 ms235 ms300 ms374 msOptimisation Time1 m 12 s8 m 30 s1h 11 m 379 h 2 m 59 s# Good Swaps2268138156Time between Two Good Swaps3 s7 s31 s209 sAverage ΔH Gain per Good Swap3 $\times 10^{-7}$2.2 $\times 10^{-8}$2.4 $\times 10^{-9}$3.7 $\times 10^{-10}$$\Delta H_{min}$1.5 $\times 10^{-5}$4.1 $\times 10^{-6}$1.04 $\times 10^{-6}$2.78 $\times 10^{-7}$

Note that matrix inversion consititues the main computational overhead of Algorithm 7. The experiments presented above have applied a standard Gaussian elimination (GE) for matrix inversion, whose complexity is $O(L^{3})$. Algorithm 7 can be sped up by (1) using a more efficient algorithm for sparse matrix inversion (SMI) and (2) recycling computations from previous matrix inversions. The table below gives the complexity of Algorithm 7 for different matrix inversion algorithms and for the classical and quantum computers.
Classical ComputerQuantum ComputerGESMI [38]SMI [40]GE [41]$O(\theta L^{3})$$O(\theta L^{2.21})$$O(\theta L\log_{2}L)$$O(\theta {\left(\log_{2}L\right)}^{3})$

The experiments have confirmed that Algorithm 7 works well and is practical for L<128. However, for a larger *L*, it grows slower and quickly becomes impractical. Optimisation of Algorithm 7 is beyond the scope of this work, and it is left for our possible future investigations. Note that the algorithm becomes very fast when it uses quantum matrix inversion.

We have taken the Calgary data corpus (see http://links.uwaterloo.ca/calgary.corpus.html, accessed on 13 April 2023) and have compressed its files using ANS instances obtained from Algorithm 7. Table 5 illustrates the results obtained.

The third column shows the sizes of files compressed using an instance of ANS with a random spread. The fourth column gives sizes of the corpus files when compressed with an instance ANS with optimised symbol spread. On average, we are getting a roughly 2.4% improvement in compression rates.

### Discussion

The main goal of compression is to reduce the redundancy of a file by encoding more frequent symbols into shorter binary strings and less frequent symbols into longer ones. Typically, for compression to work, it is necessary to describe the symbol statistics by its symbol probabilities. In this case, a file can be treated as a sequence of independent and identically distributed (i.i.d.) random variables, which correspond to the occurrence of single symbols. Clearly, such single-symbol statistics do not reflect all existing probabilistic characteristics of the file. Consequently, even the best (lossless) compression algorithm is not able to squeeze the average length of a single symbol beyond the symbol alphabet entropy. To achieve a better compression ratio, it is necessary to model file statistics by considering *N*-symbol alphabets, where N=2,3,… (see [42]). Clearly, the higher *N*, the better approximation of real statistics of the file and, consequently, better the compression rate. But the price to pay is a significant (exponential) increase in the compression complexity. We would like to emphasise that given a fixed symbol alphabet, both AC and ANS allow us to achieve a compression ratio close to the alphabet entropy. The main difference is processing speed which allows ANS to compress files for better file statistics (for *N*-symbol alphabets with higher *N*). Let us compare CPU implementations of AC and ANS. AC can reach speed of ≈200 MB/s/core while ANS achieves a speed of ≈2000 MB/s/core. For GPU implementations, ANS can be run 100 times faster than AC. This implies that, assuming the same computing resources, ANS provides better compression ratios compared to AC. This is due to the fact that ANS is faster and can apply more complex statistics for *N*-symbol alphabets (a higher *N*).

## 10. Conclusions

The work addresses an important practical problem of compression quality of the ANS algorithm. In the description of ANS, its symbol spread can be chosen at random. Each symbol spread has its own characteristic probability distribution of ANS states. Knowing the distribution, it is possible to compute the ANS compression ratio or, alternatively, its coding redundancy ΔH.

We present two algorithms that allow a user to choose symbol spreads that minimise ΔH. Algorithm 5 determines an ANS instance (its symbol spread) whose state probabilities follow the natural ANS state bias. It is fast even for $L>2^{12}$, but unfortunately, it does not provide the minimal ΔH. Algorithm 7 provides a solution. It is able to find minimal ΔH with a probability that depends on the number of random coin tosses θ.

We have conducted an experiment for L=16 that shows the behaviour of the average length of ANS encodings. Further experiments have confirmed that matrix inversion creates a bottleneck in Algorithm 7 and makes it impractical for a large *L*. An immediate remedy is the application of specialised algorithms for sparse matrix inversion, together with recycling computations from previous matrix inversions. Development of a fast version of Algorithm 7 is left as a part of our future research.

The main research challenge is, however, how to construct ANS instances in such a way that their minimum coding redundancy is guaranteed by design. It means that we have to understand the interplay between symbol spreads and their equilibrium probabilities. As ANS is also FSM, it can be visualised as a random graph whose structure is determined by symbol spread. This brings us to an interesting link between ANS and random graphs [43].

## Figures and Tables

**Table 1:** ANS for 16 states (symbol probabilities {3/16, 5/16,8/16}).

$s_{i}\backslash x_{i}$	16	17	18	19	20	21	22	23	24	25	26	27	28	29	30	31
a	$$\begin{array}{c} \left(\begin{array}{c} 22\\ 20 \end{array}\right) \end{array}$$	$$\begin{array}{c} \left(\begin{array}{c} 22\\ 01 \end{array}\right) \end{array}$$	$$\begin{array}{c} \left(\begin{array}{c} 22\\ 10 \end{array}\right) \end{array}$$	$$\begin{array}{c} \left(\begin{array}{c} 22\\ 11 \end{array}\right) \end{array}$$	$$\begin{array}{c} \left(\begin{array}{c} 25\\ 00 \end{array}\right) \end{array}$$	$$\begin{array}{c} \left(\begin{array}{c} 25\\ 01 \end{array}\right) \end{array}$$	$$\begin{array}{c} \left(\begin{array}{c} 25\\ 10 \end{array}\right) \end{array}$$	$$\begin{array}{c} \left(\begin{array}{c} 25\\ 11 \end{array}\right) \end{array}$$	$$\begin{array}{c} \left(\begin{array}{c} 18\\ 000 \end{array}\right) \end{array}$$	$$\begin{array}{c} \left(\begin{array}{c} 18\\ 001 \end{array}\right) \end{array}$$	$$\begin{array}{c} \left(\begin{array}{c} 18\\ 010 \end{array}\right) \end{array}$$	$$\begin{array}{c} \left(\begin{array}{c} 18\\ 011 \end{array}\right) \end{array}$$	$$\begin{array}{c} \left(\begin{array}{c} 18\\ 100 \end{array}\right) \end{array}$$	$$\begin{array}{c} \left(\begin{array}{c} 18\\ 101 \end{array}\right) \end{array}$$	$$\begin{array}{c} \left(\begin{array}{c} 18\\ 110 \end{array}\right) \end{array}$$	$$\begin{array}{c} \left(\begin{array}{c} 18\\ 111 \end{array}\right) \end{array}$$
b	$$\begin{array}{c} \left(\begin{array}{c} 26\\ 0 \end{array}\right) \end{array}$$	$$\begin{array}{c} \left(\begin{array}{c} 26\\ 1 \end{array}\right) \end{array}$$	$$\begin{array}{c} \left(\begin{array}{c} 28\\ 0 \end{array}\right) \end{array}$$	$$\begin{array}{c} \left(\begin{array}{c} 28\\ 1 \end{array}\right) \end{array}$$	$$\begin{array}{c} \left(\begin{array}{c} 19\\ 00 \end{array}\right) \end{array}$$	$$\begin{array}{c} \left(\begin{array}{c} 19\\ 01 \end{array}\right) \end{array}$$	$$\begin{array}{c} \left(\begin{array}{c} 19\\ 10 \end{array}\right) \end{array}$$	$$\begin{array}{c} \left(\begin{array}{c} 19\\ 11 \end{array}\right) \end{array}$$	$$\begin{array}{c} \left(\begin{array}{c} 20\\ 00 \end{array}\right) \end{array}$$	$$\begin{array}{c} \left(\begin{array}{c} 20\\ 01 \end{array}\right) \end{array}$$	$$\begin{array}{c} \left(\begin{array}{c} 20\\ 10 \end{array}\right) \end{array}$$	$$\begin{array}{c} \left(\begin{array}{c} 20\\ 11 \end{array}\right) \end{array}$$	$$\begin{array}{c} \left(\begin{array}{c} 23\\ 00 \end{array}\right) \end{array}$$	$$\begin{array}{c} \left(\begin{array}{c} 23\\ 01 \end{array}\right) \end{array}$$	$$\begin{array}{c} \left(\begin{array}{c} 23\\ 10 \end{array}\right) \end{array}$$	$$\begin{array}{c} \left(\begin{array}{c} 23\\ 11 \end{array}\right) \end{array}$$
c	$$\begin{array}{c} \left(\begin{array}{c} 16\\ 0 \end{array}\right) \end{array}$$	$$\begin{array}{c} \left(\begin{array}{c} 16\\ 1 \end{array}\right) \end{array}$$	$$\begin{array}{c} \left(\begin{array}{c} 17\\ 0 \end{array}\right) \end{array}$$	$$\begin{array}{c} \left(\begin{array}{c} 17\\ 1 \end{array}\right) \end{array}$$	$$\begin{array}{c} \left(\begin{array}{c} 21\\ 0 \end{array}\right) \end{array}$$	$$\begin{array}{c} \left(\begin{array}{c} 21\\ 1 \end{array}\right) \end{array}$$	$$\begin{array}{c} \left(\begin{array}{c} 24\\ 0 \end{array}\right) \end{array}$$	$$\begin{array}{c} \left(\begin{array}{c} 24\\ 1 \end{array}\right) \end{array}$$	$$\begin{array}{c} \left(\begin{array}{c} 27\\ 0 \end{array}\right) \end{array}$$	$$\begin{array}{c} \left(\begin{array}{c} 27\\ 1 \end{array}\right) \end{array}$$	$$\begin{array}{c} \left(\begin{array}{c} 29\\ 0 \end{array}\right) \end{array}$$	$$\begin{array}{c} \left(\begin{array}{c} 29\\ 1 \end{array}\right) \end{array}$$	$$\begin{array}{c} \left(\begin{array}{c} 30\\ 0 \end{array}\right) \end{array}$$	$$\begin{array}{c} \left(\begin{array}{c} 30\\ 1 \end{array}\right) \end{array}$$	$$\begin{array}{c} \left(\begin{array}{c} 31\\ 0 \end{array}\right) \end{array}$$	$$\begin{array}{c} \left(\begin{array}{c} 31\\ 1 \end{array}\right) \end{array}$$

**Table 2:** ANS for 16 states with state swap (symbol probabilities {3/16,5/16,8/16}).

$s_{i}\backslash x_{i}$	16	17	18	19	20	21	22	23	24	25	26	27	28	29	30	31
a	$$\begin{array}{c} \left(\begin{array}{c} 22\\ 00 \end{array}\right) \end{array}$$	$$\begin{array}{c} \left(\begin{array}{c} 22\\ 01 \end{array}\right) \end{array}$$	$$\begin{array}{c} \left(\begin{array}{c} 22\\ 10 \end{array}\right) \end{array}$$	$$\begin{array}{c} \left(\begin{array}{c} 22\\ 11 \end{array}\right) \end{array}$$	$$\begin{array}{c} \left(\begin{array}{c} 28\\ 00 \end{array}\right) \end{array}$$	$$\begin{array}{c} \left(\begin{array}{c} 28\\ 01 \end{array}\right) \end{array}$$	$$\begin{array}{c} \left(\begin{array}{c} 28\\ 10 \end{array}\right) \end{array}$$	$$\begin{array}{c} \left(\begin{array}{c} 28\\ 11 \end{array}\right) \end{array}$$	$$\begin{array}{c} \left(\begin{array}{c} 18\\ 000 \end{array}\right) \end{array}$$	$$\begin{array}{c} \left(\begin{array}{c} 18\\ 001 \end{array}\right) \end{array}$$	$$\begin{array}{c} \left(\begin{array}{c} 18\\ 010 \end{array}\right) \end{array}$$	$$\begin{array}{c} \left(\begin{array}{c} 18\\ 011 \end{array}\right) \end{array}$$	$$\begin{array}{c} \left(\begin{array}{c} 18\\ 100 \end{array}\right) \end{array}$$	$$\begin{array}{c} \left(\begin{array}{c} 18\\ 101 \end{array}\right) \end{array}$$	$$\begin{array}{c} \left(\begin{array}{c} 18\\ 110 \end{array}\right) \end{array}$$	$$\begin{array}{c} \left(\begin{array}{c} 18\\ 111 \end{array}\right) \end{array}$$
b	$$\begin{array}{c} \left(\begin{array}{c} 25\\ 0 \end{array}\right) \end{array}$$	$$\begin{array}{c} \left(\begin{array}{c} 25\\ 1 \end{array}\right) \end{array}$$	$$\begin{array}{c} \left(\begin{array}{c} 26\\ 0 \end{array}\right) \end{array}$$	$$\begin{array}{c} \left(\begin{array}{c} 26\\ 1 \end{array}\right) \end{array}$$	$$\begin{array}{c} \left(\begin{array}{c} 19\\ 00 \end{array}\right) \end{array}$$	$$\begin{array}{c} \left(\begin{array}{c} 19\\ 01 \end{array}\right) \end{array}$$	$$\begin{array}{c} \left(\begin{array}{c} 19\\ 10 \end{array}\right) \end{array}$$	$$\begin{array}{c} \left(\begin{array}{c} 19\\ 11 \end{array}\right) \end{array}$$	$$\begin{array}{c} \left(\begin{array}{c} 20\\ 00 \end{array}\right) \end{array}$$	$$\begin{array}{c} \left(\begin{array}{c} 20\\ 01 \end{array}\right) \end{array}$$	$$\begin{array}{c} \left(\begin{array}{c} 20\\ 10 \end{array}\right) \end{array}$$	$$\begin{array}{c} \left(\begin{array}{c} 20\\ 11 \end{array}\right) \end{array}$$	$$\begin{array}{c} \left(\begin{array}{c} 23\\ 00 \end{array}\right) \end{array}$$	$$\begin{array}{c} \left(\begin{array}{c} 23\\ 01 \end{array}\right) \end{array}$$	$$\begin{array}{c} \left(\begin{array}{c} 23\\ 10 \end{array}\right) \end{array}$$	$$\begin{array}{c} \left(\begin{array}{c} 23\\ 11 \end{array}\right) \end{array}$$
c	$$\begin{array}{c} \left(\begin{array}{c} 16\\ 0 \end{array}\right) \end{array}$$	$$\begin{array}{c} \left(\begin{array}{c} 16\\ 1 \end{array}\right) \end{array}$$	$$\begin{array}{c} \left(\begin{array}{c} 17\\ 0 \end{array}\right) \end{array}$$	$$\begin{array}{c} \left(\begin{array}{c} 17\\ 1 \end{array}\right) \end{array}$$	$$\begin{array}{c} \left(\begin{array}{c} 21\\ 0 \end{array}\right) \end{array}$$	$$\begin{array}{c} \left(\begin{array}{c} 21\\ 1 \end{array}\right) \end{array}$$	$$\begin{array}{c} \left(\begin{array}{c} 24\\ 0 \end{array}\right) \end{array}$$	$$\begin{array}{c} \left(\begin{array}{c} 24\\ 1 \end{array}\right) \end{array}$$	$$\begin{array}{c} \left(\begin{array}{c} 27\\ 0 \end{array}\right) \end{array}$$	$$\begin{array}{c} \left(\begin{array}{c} 27\\ 1 \end{array}\right) \end{array}$$	$$\begin{array}{c} \left(\begin{array}{c} 29\\ 0 \end{array}\right) \end{array}$$	$$\begin{array}{c} \left(\begin{array}{c} 29\\ 1 \end{array}\right) \end{array}$$	$$\begin{array}{c} \left(\begin{array}{c} 30\\ 0 \end{array}\right) \end{array}$$	$$\begin{array}{c} \left(\begin{array}{c} 30\\ 1 \end{array}\right) \end{array}$$	$$\begin{array}{c} \left(\begin{array}{c} 31\\ 0 \end{array}\right) \end{array}$$	$$\begin{array}{c} \left(\begin{array}{c} 31\\ 1 \end{array}\right) \end{array}$$

**Table 3:** Number of swaps when Searching for Optimal ANS Instances with 16 states and 3 symbols.

Average #	Min #	Max #	Min # of	Max # of
			Good Swaps	Good Swaps
≈24	4	223	4	19

**Table 4:** Cryptographic ANS.

Sender		Receiver
Secret *K*		Secret *K*
Public $P=\{p_{s}|s\in S\}$	$\overset{\;}{\to }$	Public $P=\{p_{s}|s\in S\}$
• Run Algorithm 6→$\left\{L_{s}|s\in S\right\}$		• Run Algorithm 6→$\left\{L_{s}|s\in S\right\}$
• Run Algorithm 7 with PRNG(K)		• Run Algorithm 7 with PRNG(K)
→$\left\{L_{s}^{best}|s\in S\right\}$		→$\left\{L_{s}^{best}|s\in S\right\}$
• Design encoding table for $\left\{L_{s}^{best}|s\in S\right\}$		• Build decoding table for $\left\{L_{s}^{best}|s\in S\right\}$

**Table 5:** Compression results for Calgary corpus.

File Name	Original Size in Bytes	Compressed Size (Random Spread)	Compressed Size (Optimised Spread)
bib	111,261	77,932	76,790
book1	768,771	458,920	440,678
book2	610,856	384,861	370,693
geo	102,400	69721	68,648
news	377,109	255,586	248,842
obj1	21,504	14,828	14,579
obj2	246,814	172,622	169,043
paper1	53,161	41,120	40,283
paper2	82,199	55,736	53,842
paper3	46,526	33,800	33,104
paper4	13,286	9948	9766
paper5	11,954	8954	8785
paper6	38,105	25,849	25,053
pic	513,216	120,041	115,319
progc	39,611	28,456	28,028
progl	71,646	46,669	44,905
progp	49,379	37,381	36,806
trans	93,695	74,192	73,107

## Data Availability

Draft of this paper is available at https://arxiv.org/pdf/2209.02228.pdf, accessed on 13 April 2023.
